# Targeting lactylation and the STAT3/CCL2 axis to overcome immunotherapy resistance in pancreatic ductal adenocarcinoma

**DOI:** 10.1172/JCI191422

**Published:** 2025-04-01

**Authors:** Qun Chen, Hao Yuan, Michael S. Bronze, Min Li

**Affiliations:** Department of Medicine, University of Oklahoma Health Sciences Center, Oklahoma City, Oklahoma, USA.

## Abstract

Metabolic reprogramming in pancreatic ductal adenocarcinoma (PDAC) fosters an immunosuppressive tumor microenvironment (TME) characterized by elevated lactate levels, which contribute to immune evasion and therapeutic resistance. In this issue of the *JCI*, Sun, Zhang, and colleagues identified nonhistone ENSA-K63 lactylation as a critical regulator that inactivates PP2A, activates STAT3/CCL2 signaling, recruits tumor-associated macrophages (TAMs), and suppresses cytotoxic T cell activity. Targeting ENSA-K63 lactylation or CCL2/CCR2 signaling reprograms the TME and enhances the efficacy of immune checkpoint blockade (ICB) in PDAC preclinical models. This work provides critical insights into the metabolic-immune crosstalk in PDAC and highlights promising therapeutic strategies for overcoming immune resistance and improving patient outcomes.

## Role of lactate in tumor

Lactate, once regarded as a byproduct of tumor glycolytic metabolism, is now recognized as a pivotal molecule bridging metabolic reprogramming and signal regulation, playing a crucial role in shaping and advancing the tumor microenvironment (TME) (1). In pancreatic ductal adenocarcinoma (PDAC), the dense stromal composition impedes neovascularization, creating a hypoxic and poorly perfused microenvironment that exacerbates glycolysis and lactate accumulation. This metabolic characteristic is a major contributor to the immunologically “cold” nature of PDAC (2, 3). In tumor cells, metabolism shifts toward glycolysis even in the presence of sufficient oxygen, a phenomenon known as the Warburg effect (4). This metabolic adaptation results in substantial lactate accumulation within tumor tissues. Lactate impairs the function of effector T cells and NK cells while simultaneously promoting the recruitment and polarization of myeloid-derived suppressor cells (MDSCs) and tumor-associated macrophages (TAMs), ultimately facilitating immune evasion and driving therapy resistance in PDAC (5). Beyond immune suppression, lactate serves as a metabolic substrate that fuels tumor progression by altering key signaling pathways and promoting angiogenesis, metastasis, and stromal remodeling within the TME (6). More specifically, lactate modulates the acidic microenvironment via monocarboxylate transporters (MCTs), enhancing angiogenesis, extracellular matrix remodeling, and immune escape (7). Additionally, lactate acts as a ligand for the GPR81 receptor, activating downstream signaling pathways that further enhance tumor cell invasiveness and adaptability (8). Lactate not only alters metabolism, but also regulates gene expression through lysine lactylation, an epigenetic modification, as demonstrated by Zhang et al., who showed that histone lactylation upregulates Arg1 expression, driving M2 macrophage polarization and promoting tumor immune evasion (9). However, targeting histone lactylation is limited by certain technological challenges, including lack of cell-type specificity, potential for divergent histone protein functions across different cell types, and competition among multiple epigenetic modifications on the same histone. In contrast, nonhistone lactylation may hold greater potential, particularly in the regulation of transcription factors and metabolic enzymes involved in nonclassical signaling pathways. Yang et al. recently demonstrated that K28 lactylation of AK2 disrupts energy metabolism and enhances malignancy in hepatocellular carcinoma, further expanding our understanding of lactate’s role in shaping the TME (10). These findings highlight nonhistone lactylation as a promising regulatory mechanism that may provide a more precise therapeutic target for cancer metabolism and immune modulation (11).

Taken together, further investigation into the diverse regulatory roles of lactate in PDAC, along with the development of targeted strategies to modulate lactate metabolism, holds great promise for enhancing therapeutic efficacy and improving patient outcomes.

## Lactylation drives immunosuppressive TME in PDAC

Building on the established role of lactate in modulating the TME, increasing evidence highlights its impact on immune cell reprogramming and immunotherapy resistance (12). In this issue of the *JCI*, Sun, Zhang, and colleagues (13) leveraged fluorine-18 fluorodeoxyglucose (^18^F-FDG) PET-CT imaging, a clinically established tool for quantifying glycolytic activity, to noninvasively infer lactate accumulation in PDAC tissues. By correlating regions of high FDG uptake with the immunosuppressive TME, they revealed a functional link between elevated glycolytic tumors and therapy-resistant microenvironments (13). The authors demonstrated that tumor-derived lactate accumulates in the TME, driving lactylation, which is most prominent in TAMs. Notably, the elevated lactylation in TAMs was not driven by increased glycolysis within these cells but rather by enhanced lactate uptake from the TME. Single-cell RNA sequencing analysis revealed that macrophages in highly glycolytic tumors were reprogrammed into a protumorigenic phenotype, while CD8^+^ T cells displayed a trend toward exhaustion (13). This dual shift underscores the role of tumor glycolysis in fostering an immunosuppressive TME, ultimately leading to immune evasion and therapy resistance.

Further, lactylation levels in tumor tissues demonstrated remarkable specificity (86.7%), sensitivity (92.9%), and an area under the receiver operating characteristic (ROC) curve (AUC) of 95.59 for predicting immunotherapy outcomes, surpassing commonly used indicators such as PD-L1 expression, tumor mutation burden (TMB), and microsatellite instability (MSI) (13–16). If validated in larger-scale studies, lactylation levels could serve as a highly promising prognostic biomarker for guiding immunotherapy in PDAC.

**Figure 1:**
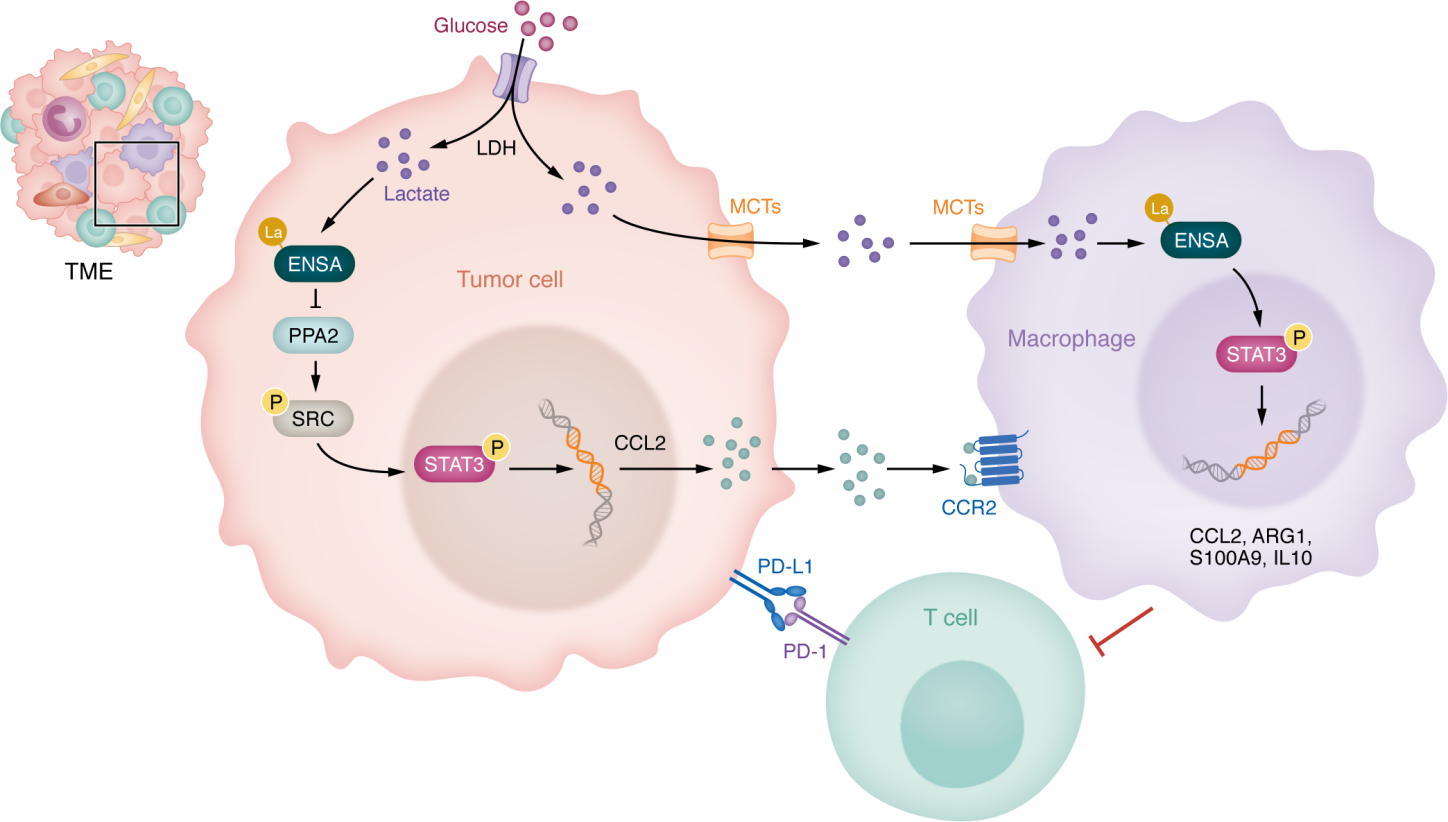
Lactate-induced ENSA-K63 lactylation drives the formation of an immunosuppressive microenvironment in PDAC. In PDAC cells, lactate, generated by LDH during glycolysis, induces ENSA-K63 lactylation (la), which inhibits PP2A activity and sustains SRC phosphorylation. This activation triggers STAT3 phosphorylation, driving the transcriptional upregulation of CCL2. CCL2 recruits macrophages via CCR2. Separately, extracellular lactate secreted by tumor cells through MCTs is taken up by macrophages, further reprogramming them through the ENSA/SRC/STAT3/CCL2 axis and amplifying the expression of genes encoding immunosuppressive factors (including CCL2, ARG1, S100A9, and IL10). These processes establish an immunosuppressive microenvironment and promote resistance to T cell–mediated antitumor immunity and PD-1–mediated immunotherapy.

By integrating these findings, Sun, Zhang, and colleagues identified a key mechanistic pathway in which elevated tumor glycolysis recruits monocytes and macrophages through the ENSA-K63la/SRC-pS12/STAT3-pY705/CCL2 axis. Increased glycolysis leads to lactate accumulation, driving lactylation at the K63 site of the ENSA protein (ENSA-K63la), a form of nonhistone lactylation in PDAC. This modification inhibits PP2A activity, resulting in the hyperphosphorylation of SRC and STAT3. Consequently, the secretion of CCL2, a chemokine that recruits monocytes and macrophages into the TME, is upregulated. These recruited TAMs then adopt a protumor phenotype, further reinforcing the immunosuppressive TME (Figure 1). Interestingly, CD8^+^ T cells exhibit differential responses to lactate stimulation. While most CD8^+^ T cells are unable to upregulate lactylation modifications in response to exogenous lactate, naive CD8^+^ T cells (CD45^+^CD3^+^CD8^+^CD25^–^) displayed an increase in overall intracellular lysine lactylation levels when exposed to tumor-secreted lactate. This contrasting behavior suggests that activated CD8^+^ T cells with elevated glycolysis-derived intracellular lactate may be resistant to further lactate accumulation (13). This finding provides an important mechanistic insight into immunotherapy resistance in PDAC and highlights the pivotal role of dysregulated glucose metabolism in driving immune suppression. Clinically, PDAC patients with high tumor glycolytic activity exhibited poor responses to immunochemotherapy and reduced survival rates (13).

Moreover, Sun, Zhang, and colleagues developed a peptide inhibitor specifically targeting ENSA lactylation, named K63-pe-3, which effectively blocked this signaling pathway in vivo and enhanced sensitivity to immunotherapy in murine models (13). This cascade of findings provides a comprehensive, mechanistic explanation for immunotherapy resistance in PDAC, while also offering promising therapeutic avenues.

To translate the Sun, Zhang, and colleagues discovery into clinical applications, the authors proposed three potential therapeutic approaches to overcome immunotherapy resistance in PDAC: a KRAS-G12D mutation inhibitor, the targeted cell-penetrating peptide K63-pe-3, and a CCR2 inhibitor (13). While these approaches have demonstrated antitumor effects in preclinical models, further validation is required to evaluate their clinical applicability. Additionally, the CCL2/CCR2 axis plays multifaceted roles in the TME, not only promoting macrophage recruitment but also potentially influencing the activity of other immune cell populations (17, 18). To enhance therapeutic efficacy, dual-targeting agents against both CCL2 and CCR2, or their combination with established immune checkpoint inhibitors (e.g., PD-1/PD-L1 inhibitors), could represent promising strategies. STAT3 is a well-established oncogenic molecule, with inhibitors such as OPB-31121 and TTI-101 demonstrating safety and potential efficacy in phase I clinical trials for advanced solid tumors, including PDAC (19, 20). Additionally, several ongoing clinical trials are evaluating STAT3 inhibitors in cancer treatment, including VVD-130850 (ClinicalTrials.gov NCT06188208) for advanced solid and hematologic malignancies, silibinin (NCT05689619) for preventing brain metastases in non–small cell lung cancer and breast cancer, WP1066 (NCT05879250) in combination with radiotherapy for glioblastoma, and danvatirsen (NCT05986240) in combination with venetoclax for high-risk myelodysplastic syndrome and acute myeloid leukemia. Future research could focus on leveraging STAT3 inhibitors to disrupt the macrophage polarization axis, exploring their potential to modulate the immunosuppressive microenvironment in PDAC and offering valuable insights for optimizing combination therapy strategies. Despite the need for further validation, these findings hold important theoretical and clinical implications, paving the way for strategies that enhance cancer immunotherapy and improve patient outcomes in PDAC.

## Conclusions and future directions

Sun, Zhang, and colleagues demonstrated a critical role of lactate accumulation and enhanced protein lactylation in remodeling the immune microenvironment and driving immunotherapy resistance in PDAC (13). The study uncovers a glycolysis-dependent ENSA-K63 lactylation/SRC-pS12/STAT3-pY705 signaling axis that promotes CCL2 secretion, leading to increased macrophage infiltration and suppressed cytotoxic T cell activity, ultimately contributing to immunotherapy resistance. A key highlight of this study is the discovery that lactate derived from tumor glycolytic metabolism accumulates within cancer cells, where tumor-derived lactate promotes CCL2 expression through STAT3 activation, thereby inducing recruitment and polarization of TAMs toward an M2-like phenotype in PDAC. Another key finding of this study is the identification of the unique and pivotal role of lactate accumulation in shaping the immunosuppressive environment and driving immunotherapy resistance in PDAC. Tumor-derived lactate acts as a primary driver in establishing the immunosuppressive microenvironment and promoting resistance to immunotherapy. While the lactylation-targeted therapeutic strategies proposed by the authors have demonstrated promising therapeutic potential, further investigations, along with validation in larger multicenter clinical cohorts, are essential to elucidate the underlying mechanisms and enhance their clinical applicability.

In summary, Sun, Zhang, and colleagues (13) provide important insights into metabolism-immune interactions in PDAC, highlighting a promising pathway for developing innovative lactate-targeted therapies and combination strategies to address immune evasion and immunotherapy resistance in this malignancy.

## Version 1 04/01/2025

Electronic publication
